# IL-1β Blockade Attenuates Thrombosis in a Neutrophil Extracellular Trap-Dependent Breast Cancer Model

**DOI:** 10.3389/fimmu.2019.02088

**Published:** 2019-09-04

**Authors:** Tainá Gomes, Carolina B. S. Várady, André L. Lourenço, Daniella M. Mizurini, Araci M. R. Rondon, Ana C. Leal, Barbara S. Gonçalves, Dumith Chequer Bou-Habib, Emiliano Medei, Robson Q. Monteiro

**Affiliations:** ^1^Institute of Medical Biochemistry Leopoldo de Meis, Federal University of Rio de Janeiro, Rio de Janeiro, Brazil; ^2^Laboratory on Thymus Research, Oswaldo Cruz Institute/Fiocruz, Rio de Janeiro, Brazil; ^3^National Institute of Science and Technology on Neuroimmunomodulation, Rio de Janeiro, Brazil; ^4^Carlos Chagas Filho Biophysics Institute, Federal University of Rio de Janeiro, Rio de Janeiro, Brazil

**Keywords:** cancer, IL-1β, G-CSF, neutrophil extracellular trap (NET), thrombosis

## Abstract

Cancer patients are at increased risk of developing thrombosis, comorbidity that has been associated with increased neutrophil counts and the formation of neutrophil extracellular traps (NETs). Interleukin-1β (IL-1β) modulates the expression of granulocyte colony-stimulating factor (G-CSF), a cytokine that promotes cancer-associated neutrophilia and NET generation. Herein, we combined a murine breast cancer model with a flow-restriction thrombosis model to evaluate whether the IL-1β blockade could interfere with cancer-associated thrombosis. Mice bearing metastatic 4T1 tumors exhibited high neutrophil counts as well as elevated expression of G-CSF and IL-1β in their tumors. On the other hand, mice bearing non-metastatic 67NR tumors showed no elevation in neutrophil counts and displayed low expression levels of G-CSF and IL-1β in their tumors. 4T1 tumor-bearing mice but not 67NR tumor-bearing mice exhibited a NET-dependent prothrombotic state. Pharmacological blockade of IL-1 receptor (IL-1R) decreased the primary growth of 4T1 tumors and reduced the systemic levels of myeloperoxidase, cell-free DNA (cfDNA) and G-CSF, without interfering with the neutrophil counts. Most remarkably, the blockade of IL-1R abolished the prothrombotic state observed in 4T1 tumor-bearing mice. Overall, our results demonstrate that IL-1β might be a feasible target to attenuate cancer-associated thrombosis, particularly in cancer types that rely on increased G-CSF production and involvement of NET formation.

## Introduction

The incidence of thrombosis in patients with cancer is higher than that in a general population free from tumor burdens (1, 2). Cancer-associated thrombosis is usually correlated with a worse prognosis and is the second leading cause of death in cancer patients. In this context, the incidence, prevalence, and treatment of cancer-associated thrombosis have prompted multiple research campaigns worldwide, but the mechanisms that allow this phenomenon to occur have yet to be fully elucidated. In an attempt to identify the cancer patients who are at the highest risk of developing a thrombotic event, multiple risk factors and biomarkers have been evaluated (1, 3–6). Among those, leukocytosis has become an event well-correlated with venous thrombosis (7, 8). There is compelling evidence that neutrophil extracellular traps (NETs) are important players in thrombus formation (9, 10). NETs comprise a molecular trap formed by DNA, histones, and proteins derived from the neutrophil granules that were first described as a defense mechanism against microbes (11). Subsequent studies have demonstrated that NETs display a number of thrombogenic properties, including the ability to initiate the intrinsic pathway of coagulation, serving as a scaffold for the adherence of platelets and red blood cells, to degrade natural coagulation inhibitors, and finally, to exert antifibrinolytic effects (12). Therefore, NET formation is critical for venous thrombus formation in mouse models of thrombosis (13, 14). The involvement of NETs in cancer-associated thrombosis has been proposed (15, 16), and the enhanced generation of NETs parallels the establishment of a prothrombotic state in animal models (17–19). Moreover, recent studies have associated the plasma elevation of NET generation markers with thrombotic manifestations in cancer patients (20).

Granulocyte colony-stimulating factor (G-CSF) is a cytokine that stimulates the bone marrow to produce granulocytes and is the main regulator of neutrophil generation and differentiation *in vivo* (21, 22). Several protumorigenic effects have been attributed to G-CSF, which might be overproduced during tumor progression (23, 24). In this context, a recent study demonstrated that G-CSF expression correlates with poor survival in human triple-negative breast cancer (25). The protumorigenic effects of G-CSF are driven, at least in part, by its ability to facilitate NET formation (15, 26). Interestingly, increased G-CSF levels correlate with NET-associated thrombosis in animal models and in cancer patients (17–19, 27).

Tumor-derived interleukin-1β (IL-1β) has recently been implicated in the systemic increase in G-CSF expression by supporting IL-17 expression by gamma delta T cells (28). Pharmacological or genetic inhibition of IL-1β reduces tumor growth and metastasis in murine and human breast cancer models (29, 30). Furthermore, treatment with a recombinant IL-1 receptor (IL-1R) antagonist alters the infiltrating immune cell composition of the tumor microenvironment (30). In the present study, we combined a well-established murine breast cancer model with a flow-restriction deep vein thrombosis (DVT) model (31) to interrogate whether the inhibition of IL-1β could attenuate cancer-associated thrombosis. Our data show that mice bearing tumors with elevated expression of G-CSF and IL-1β exhibit increased neutrophil counts and rely on the establishment of a NET-dependent prothrombotic state. Remarkably, blockade of IL-1R diminished G-CSF levels, lowered NET formation markers and significantly reduced the prothrombotic state in tumor-bearing mice. Overall, our results demonstrate that targeting IL-1β may attenuate cancer-associated thrombosis, particularly in cancer types that rely on increased G-CSF production and involvement of NET formation.

## Materials and Methods

### Cell Lines and Cell Culture

The cell lines 4T1 and 67NR, which originated from spontaneous breast tumors in BALB/c mice (32), were purchased from the Karmanos Cancer Institute (MI, USA). The cells were maintained in high glucose Dulbecco's-modified Eagle medium (DMEM, Gibco, MA, USA) supplemented with 2.4 g/L HEPES, 3.7 g/L sodium bicarbonate, 100 U/mL penicillin (Gibco, MA, USA), 100 μg/L streptomycin (Gibco, MA, USA) and 10% FBS (fetal bovine serum, Cultilab, SP, Brazil) at 37 °C in a humidified atmosphere of 5% CO_2_. Cells were kept in culture for no more than five passages before being inoculated into mice.

### Animals

This investigation was conducted in accordance with ethical standards and according to the Declaration of Helsinki and national and international guidelines and has been approved by the institutional animal care and use committee (IACUC) of the Federal University of Rio de Janeiro. The animal procedures were approved by the IACUC under protocol number CEUA102/16. For each experiment, the animals were anesthetized with subcutaneous xylazine (16 mg/kg, Syntec, SP, Brazil) followed by ketamine (100 mg/kg, Syntec, SP, Brazil).

### Tumor Induction

Female BALB/c mice (8–10 weeks old) were inoculated with 5 × 10^5^ 4T1 or 67NR cells (in 100 μL phosphate-buffered saline) in the fourth mammary fat pad. The animals were monitored every other day for tumor growth, and the tumors were measured with calipers. The tumor volume was calculated using the formula V = (l × w^2^)/2, where l is the length and w is the width. The animals were housed under controlled conditions for both temperature (24 ± 1°C) and light (12 h of light starting at 7:00 a.m.).

### Blood Cell Analysis

The mice were anesthetized as described above, and the blood was collected into EDTA buffer (1.8 mg/mL) by cardiac puncture. The blood cell count was determined using a CELL-DYN 3500 hematology analyzer (Abbott Diagnostics, IL, USA).

### RNA Isolation and Real-Time Quantitative (Qrt-PCR)

RNA was prepared from cultured cells (5 × 10^5^) or from tumor tissue (100 mg) using TRIzol Reagent (Invitrogen, CA, USA). The quantification of the RNA was performed using a NanoDrop Lite (Thermo Fisher Scientific, MA, USA) measuring the absorbances at 260/280 nm. The mRNA purity was evaluated, and a ratio above 1.8 was required for the mRNA to be used. cDNA was synthesized using a SuperScript II Reverse Transcriptase kit (Thermo Fisher Scientific, MA, USA). qRT-PCR was performed with SYBR green Master Mix (Thermo Fisher Scientific, MA, USA) on a PCR *Fast Real-Time* instrument (Applied Biosystems 7900HT, CA, USA). The expression of each gene was normalized to that of β-*actin*, the endogenous control. Relative gene expression was analyzed using the ΔΔCt approach. The primers used were as follows: *G-CSF*: F:GCAAGTGAGGAAGATCCA; R:CTAGAGCAGCCACTCAGG; *IL-1*β*;* F:AAATGCCACCTTTTGACAGTGATG; R:GCTCTTGTTGATGTGCTGCTG; and β*-actin* F:ATGGTGGGAATGGGTCAGAAG; R:TTCTCCATGTCGCAGTTG.

### Quantification of Plasma Cell-Free DNA Levels

The cell-free DNA (cfDNA) levels in the plasma were quantified using a Quant-it Picogreen dsDNA kit (Thermo Fisher Scientific, MA, USA) according to the manufacturer's instructions. Succinctly, 5 μL mouse plasma from control or tumor-bearing mice was diluted in 45 μL TE buffer (200 mM Tris-HCl, 20 mM EDTA, pH 7.5), and 50 μL Picogreen was added per well in a Luminescence test 96-well plate. Fluorescence was measured in an automated spectrofluorimetric reader (Spectra Max Paradigm; Molecular Devices, CA, USA) set to 485 nm for excitation and 535 nm for emission.

### Quantification of G-CSF and Myeloperoxidase in the Plasma

G-CSF and myeloperoxidase were quantified with specific ELISA kits. Plasma levels of G-CSF were determined using a Mouse G-CSF DuoSet ELISA (DY414- R&D System, MN, USA) and myeloperoxidase levels were quantified with a Mouse Myeloperoxidase ELISA (DY3667- R&D System, MN, USA). The analyses were performed according to the manufacturer's instructions.

### Quantification of DNA-Elastase Complex in the Plasma

The plasma level of DNA-elastase was measured by using a modified sandwich ELISA as previously described (33). In brief, a 96-well plate was coated with 5 μg/mL anti-NE monoclonal antibody (ab68672, Abcam, MA, USA), at 4°C overnight. On the next day, the plate was washed three times with PBS and blocked with 1% BSA in PBS, for 1 h, at room temperature. After three washes with PBS, 50 μL samples (plasma diluted 1:1 in PBS) were added to the wells and incubated at room temperature for 1 h under gentle agitation, and kept at 4°C overnight. The plate was washed with 0.05% Tween in PBS three times before the addition of 50 μL anti-dsDNA HRP-conjugated antibody (1:1,000, #21227778, ImmunotTools, Germany). The plate was incubated for 2 h under gentle agitation at room temperature and washed three times prior to the addition of 50 μL peroxidase substrate TMB Single Solution (Life Technologies, MD, USA). The reaction was stopped after 30 min incubation, by adding an equal amount of 2 N H_2_SO_4_, and the absorbance was measured at 450 nm using a microplate reader.

### Western Blot Analysis

The tumor tissue samples (200 mg) were lysed using an Ultra Turrax T25 homogenizer (IKA Labortechnik, province of Breigaus-Hochschwarzwald, Germany) with a solution containing a protease (# 11697498001, Roche, BS, Switzerland) and phosphatase (# 04906845001, Roche, BS, Switzerland) inhibitor cocktail. The supernatants were collected, and the protein concentrations were determined using the modified Lowry method (Bio-Rad DC Protein Assay; Bio-Rad Laboratories, CA, USA). A total of 30 μg protein was separated by 12% SDS-PAGE and then transferred to a PVDF blotting membrane (Amersham Hybond TM, GE Healthcare, IL, USA). The membrane was blocked with 5% BSA (bovine serum albumin, Sigma-Aldrich, MO, USA) and then incubated with the primary antibody rabbit polyclonal anti-histone H3 (citrulline R2 + R8 + R17) (1:1,000; ab5103, Abcam, MA, USA) or rabbit monoclonal anti-β-actin (1:1,000, D6A8, #8457, Cell Signaling, MA, USA) overnight at 4°C. The membranes were then incubated with a goat anti-rabbit IRDye®680LT secondary antibody (1:10,000, LI-COR, NE, USA) for 1 h. The bands were visualized using an Odyssey Infrared Imaging System (LI-COR, NE, USA). Quantification data were normalized to β-actin expression.

### Flow Cytometry

Tumor tissue (200 mg) was mechanically disaggregated with scissors and flushed through a 70 μm cell strainer (#431751, Corning, NY, USA). Cells were stained with a rat monoclonal antibody against Ly6G conjugated with FITC (1 μL per 10^6^ cells, ab25024, Abcam, MA, USA), rat polyclonal antibody to CD11b conjugated with APC (1:200, #101212, Biolegend, CA, USA) and rabbit monoclonal antibody to VEGF-A (1:500, ab52917, Abcam, MA, USA). The samples were incubated with the primary antibodies at specific concentrations for each antibody for 60 min on ice. An anti-rabbit IgG conjugated with PE antibody (1:200, sc-3739, Santa Cruz Biotechnology, TX, USA) was used as a secondary antibody for VEGF-A staining. The acquisition procedure was performed using a FACSVerse instrument (BD Biosciences, NJ, USA).

### CFSE Assay

A total of 2 × 10^6^ 4T1 cells were trypsinized and labeled with 5 μM CFSE (5(6)-carboxyfluorescein diacetate N-succinimidyl ester, Life Technologies, MA, USA). Then, 5 × 10^5^ labeled cells were plated per well in 6-well plates. After 24 h of serum starvation, 100 μg anakinra was added to each well. Every 24 h, the cells were trypsinized and resuspended in PBS. The acquisition procedure was performed using a FACSVerse flow cytometer (BD Biosciences, NJ, USA).

### MTT Assay

A cell viability assay was performed by incubating the cells with the salt MTT (3-(4,5-dimethylthiazol-2-yl)-2,5-diphenyltetrazolium bromide) (M2128, Sigma-Aldrich, MO, USA). A total of 10^4^ 4T1 cells were plated per well into 96-well plates in 200 μL per well. Three parallel wells were designated for each experimental group. After 24 h of serum starvation, different concentrations of anakinra were added to each well. Every 24 h, 20 μL MTT (5 mg/mL) was added to a subset of the wells and incubated for 3 h before the culture medium was discarded, and the reaction was terminated by adding 100 μL DMSO (D8418, Sigma-Aldrich, MO, USA). The absorbance was determined at 538 nm using a Spectra Max Paradigm spectrophotometer (Molecular Devices, CA, USA).

### Mouse Model of Flow Restriction in the Inferior Vena Cava (IVC)

Mice were anesthetized as described above. A median laparotomy was performed, and the IVC was exposed. We positioned a space holder (0.3 mm insulin needle) on the outside of the vessel, and we placed a permanent narrowing ligature (Mononylon 6-0 Ethicon, NJ, USA) exactly below the left renal vein. In the end, the needle was removed to avoid complete vessel occlusion. After the time indicated in each experiment, the thrombus that formed below the suture in the caudal direction was removed and weighed (14, 31).

### DNase I, GSK484, Anakinra, and MCC950 Treatment

Tumor-bearing or tumor-free mice were injected intravenously with 50 μg DNase I (Pulmozyme® Genentech, CA, USA) or 10 mg/kg GSK484 (Aobious, MA, USA) in a final volume of 50 μL diluted in injection water. The injections were performed via the retro-orbital vein before the start of the DVT procedure. Treatment with anakinra (10 mg/kg/day, Kineret® SOBI, Sweden) or MCC950 (20 mg/kg/day, Aobious, MA, USA) was performed every 8 days between day 14 and day 21 after inoculation. The injections were performed subcutaneously.

### Statistical Analysis

The results are expressed as the mean ± SD or are depicted as scatter plots. Statistical analyses were performed using GraphPad Prism 5 software (GraphPad Software, CA, USA). One-way analysis of variance (ANOVA) followed by Tukey's *post-hoc* test was used for comparisons between the test groups. If only two groups were compared, an unpaired 2-tailed Student's *t-*test was applied. Differences were considered significant when *P* < 0.05.

## Results

### 4T1 Cells Exhibit Higher Levels of IL-1β, G-CSF, and NET Formation Than 67NR Cells in the Tumor Microenvironment

**Figure 1:**
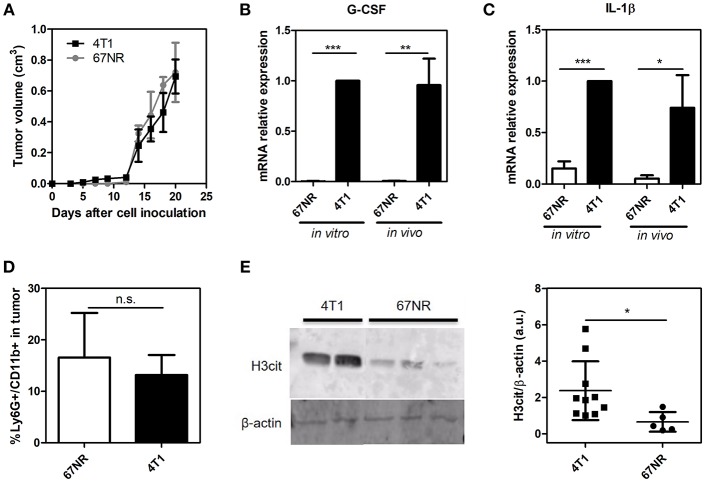
4T1 cells exhibit higher levels of IL-1β, G-CSF and NET formation than 67NR cells in the tumor microenvironment. 4T1 and 67NR tumor cells (5 × 10^5^) were orthotopically injected into the mammary fat pads of female BALB/c mice. **(A)** The sizes of both the 4T1 (■, *n* = 10) and 67NR (•, *n* = 3) tumors were monitored continuously over 21 days through measurements every 2 days. The expression of the *G-CSF* **(B)** and *IL-1*β **(C)** genes was evaluated by qRT-PCR using 4T1 (*n* = 3) and 67NR (*n* = 3) tumor cells or tumor lysates from 4T1 (*n* = 6) and 67NR (*n* = 6) tumor-bearing mice. β*-actin* was used as a reference gene for normalization. The relative expression level of each mRNA was calculated using the ΔΔCT method. **(D)** The infiltration of neutrophils in 4T1 (*n* = 6) and 67NR (*n* = 3) tumors was analyzed 21 days after cell inoculation. The tumors were dissociated into single cells and assessed for Ly6G^+^ CD11b^+^ cells via flow cytometry. **(E)** To evaluate the presence of NETs in the tumor microenvironment, the levels of citrullinated histone (H3cit) were investigated by Western blotting proteins from 4T1 (*n* = 10) and 67NR (*n* = 5) tumors. The right panel shows the quantification of H3cit protein levels normalized to β-actin levels. For the original uncropped western blot data (see Supplementary Information). Each dot represents one individual mouse. Values represent the mean ± SD. ^*^P < 0.05, ^**^P = 0.01, and ^***^P = 0.001; n.s., non-significant, unpaired two-tailed Student's *t*-test.

We employed a well-established mouse mammary tumor model comprised of two isogenic tumor cell lines: 67NR and 4T1. Derived from a single mammary tumor, both cell lines formed orthotopic tumors when inoculated in the mammary fat pad of female BALB/c mice. However, while 4T1 cells are highly metastatic, 67NR cells are unable to disseminate to distant sites (32). Despite these traits, the primary tumor growth rates of 4T1 and 67NR tumors did not present significant differences during the observed period of this study (21 days), as shown in Figure 1A. 4T1 tumors have been described as potent producers of G-CSF, a growth factor that displays several biological effects and has been associated with the metastatic potential of 4T1 cells (34, 35). Accordingly, we observed that both plated 4T1 cells and their derived solid tumors exhibited significantly higher *G-CSF* gene expression levels compared to 67NR cultured cells and tumors, respectively (Figure 1B). Recent studies have implicated interleukin IL-1β in the systemic upregulation of G-CSF in mouse models of breast cancer (28). In this context, qRT-PCR analysis of both cultured and implanted 67NR and 4T1 cells demonstrated dramatic differences in *IL-1*β gene expression (Figure 1C). Despite the significant differences in the G-CSF and IL-1β levels between the 4T1 and 67NR tumor microenvironments, no significant differences in neutrophil (Ly6G^+^ CD11b^+^ cells) infiltration were observed (Figure 1D). The secretion of G-CSF by the primary tumor has been correlated with the formation of NETs in the tumor microenvironment (36). Therefore, we observed that 4T1 tumors presented significantly higher levels of citrullinated histone, a biomarker frequently associated with NET formation, within the tumor microenvironment compared to their 67NR counterparts (Figure 1E and Supplementary Figure 1).

### 4T1 Tumor-Bearing Mice but Not 67NR-Bearing Mice Exhibit Neutrophilia and a NET-Dependent Prothrombotic State

**Figure 2:**
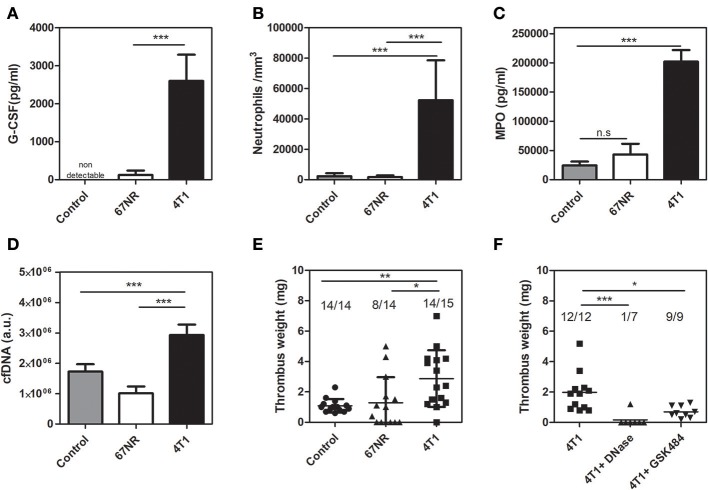
4T1 tumor-bearing mice but not 67NR tumor-bearing mice exhibit neutrophilia and a NET-dependent prothrombotic state. **(A)** Systemic levels of G-CSF in control (gray bars, *n* = 5), 67NR (open bars, *n* = 3) and 4T1 (black bars, *n* = 5) tumor-bearing mice were assessed by a specific ELISA kit as described in section Materials and Methods. **(B)** Neutrophil counts in control (gray bars, *n* = 12), 67NR (open bars, *n* = 5) and 4T1 (black bars, *n* = 11) tumor-bearing mice were evaluated as described in section Materials and Methods. Two systemic markers of NETs, MPO **(C)** and cfDNA **(D)**, were analyzed in control (gray bars, *n* = 5), 67NR (open bars, *n* = 3) and 4T1 (black bars, *n* = 6) tumor-bearing mice as described in section Materials and Methods. For systemic analysis, the blood was collected 21 days after the tumor cell inoculation. **(E)** Venous thrombosis was evaluated with stasis of the inferior vena cava for 6 h in control (•, *n* = 14), 67NR (▴, *n* = 14), and 4T1 (■, *n* = 15) tumor-bearing mice. **(F)** Alternatively, 4T1 tumor-bearing mice (■, *n* = 12) were treated with DNase I (50 μg, i.v.) (▴, *n* = 7) or GSK484 (10 mg/kg, i.v.) (▾, *n* = 9) just before performing the procedure to induce stasis of the inferior vena cava for 3 h. Each dot represents one individual mouse. Values represent the mean ± SD. ^*^P < 0.05, ^**^P = 0.01, and ^***^P = 0.001; n.s., non-significant, analysis of variance (ANOVA) followed by Tukey's *post-hoc* test.

As observed with cultured cells and primary tumors, animals inoculated with either 4T1 or 67NR cells greatly differed in their systemic G-CSF levels (Figure 2A). As a consequence, 4T1 tumor-bearing mice but not 67NR tumor-bearing mice exhibited a dramatic increase in peripheral neutrophil counts (Figure 2B). Other hematological changes, including elevated monocyte, lymphocyte and platelet counts, were typically observed in 4T1 tumor-bearing mice but not in 67NR-bearing mice (Supplementary Table 1). The elevation of systemic G-CSF levels has been correlated with increased NET formation (17–19). In this context, 4T1 tumor-bearing mice exhibited increased circulating levels of myeloperoxidase (Figure 2C) and cfDNA (Figure 2D), which are considered indirect biological markers of NET formation. We further employed a DVT model based on the partial stenosis of the inferior vena cava (14, 31) to evaluate the prothrombotic state in tumor-bearing mice. The results obtained with this DVT model revealed that 4T1 tumor-bearing mice developed significantly heavier thrombi in comparison with mice bearing 67NR tumors (2.87 ± 0.48 vs. 1.29 ± 0.45 mg, respectively) or with the tumor-free naive controls (1.09 ± 0.12 mg) after 6 h of flow restriction (Figure 2E). No significant differences were observed between the control and 67NR tumor-bearing mice. We previously observed that NET formation is essential for the prothrombotic state in 4T1 tumor-bearing mice in other thrombosis models (18). In accordance with our previous observations, thrombus formation in the DVT model was highly dependent on NETs since prior treatment with DNase I, which degrades NETs, significantly attenuated thrombus formation after 3 h of flow restriction (Figure 2F). Treatment with a single dose of DNase I was equally efficient in reducing thrombus formation in the 4T1 model after 6 h of flow restriction (Supplementary Figure 2). Peptidylarginine deiminase 4 (PAD4), a nuclear enzyme that converts specific arginine residues to citrulline in histone tails, plays a major role in NET formation (37) and DVT development in mice (38). Accordingly, Figure 2F shows that GSK484, a reversible inhibitor of PAD4 (39), significantly attenuated thrombus formation in 4T1 tumor-bearing mice.

### The IL-1 Receptor Antagonist, Anakinra, Reduces Tumor Growth in 4T1 Tumor-Bearing Mice but Not in 67NR Tumor-Bearing Mice

**Figure 3:**
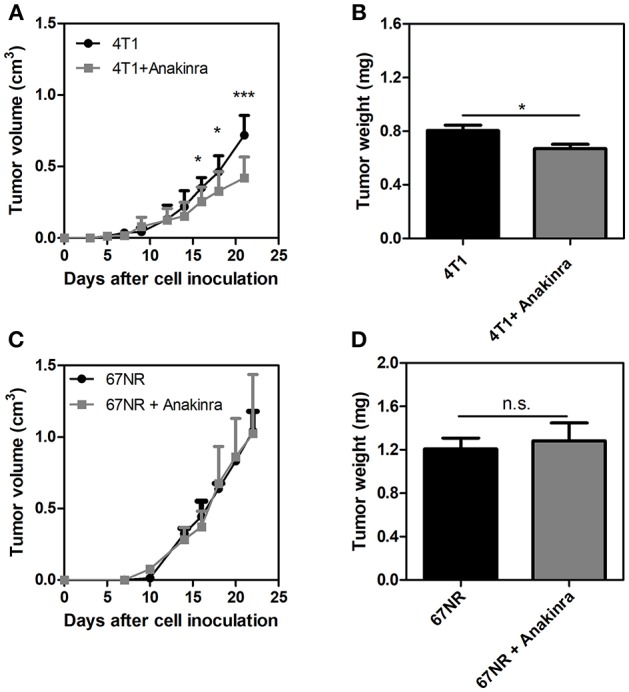
The IL-1R antagonist anakinra reduces tumor growth in 4T1 tumor-bearing mice but not 67NR-bearing mice. Both 4T1 tumor-bearing mice **(A)** and 67NR tumor-bearing mice **(C)** received treatment with anakinra (10 mg/kg/day, s.c.) for 8 days between day 14 and day 21 after the tumor cell inoculation. The graphs show the tumor growth and the weight of the tumor **(B,D)** assessed 21 days after the inoculation. Values represent the mean ± SD. ^*^P < 0.05 and ^***^P = 0.001; n.s., non-significant, unpaired two-tailed Student's *t*-test.

**Figure 4:**
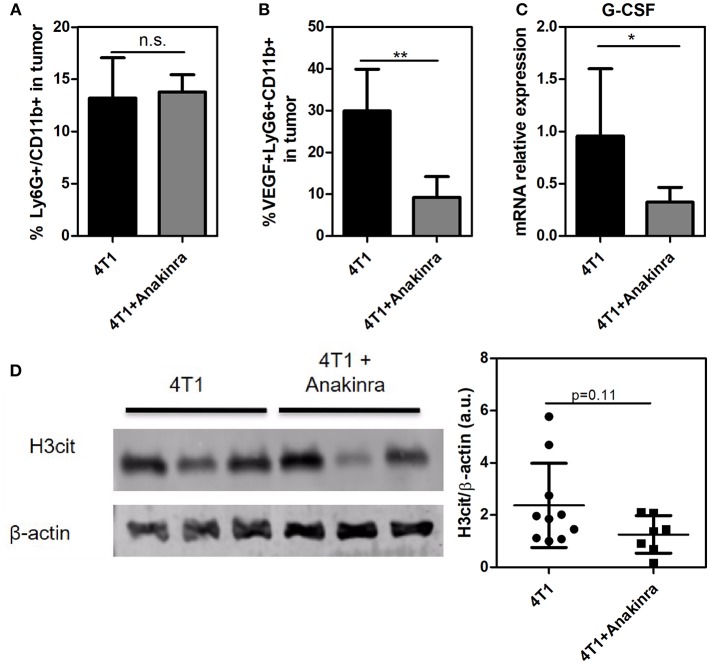
Impact of blocking IL-1R in the 4T1 tumor microenvironment. Both 4T1 and 67NR tumor-bearing mice received anakinra (10 mg/kg/day, s.c.) for 8 between day 14 and day 21 after the tumor cell inoculation. **(A)** The infiltration of neutrophils into 4T1 (*n* = 6) and anakinra-treated 4T1 (*n* = 6) tumors was analyzed 21 days after the cell inoculation. The tumors were dissociated into single cells and assessed for Ly6G^+^ CD11b^+^ cells via flow cytometry. **(B)** The expression of VEGF in tumor-associated neutrophils in the 4T1 tumor (black bars, *n* = 4) or anakinra-treated 4T1 (gray bars, *n* = 6) tumors was assessed by VEGF expression in Ly6G^+^ CD11b^+^ cells. **(C)** The expression of the *G-CSF* gene was analyzed by qRT-PCR in tumor lysates from 4T1 (black bar, *n* = 6) and anakinra-treated 4T1 (gray bar, *n* = 6) tumors. β*- actin* was used as a reference gene for normalization. The relative expression level of the mRNA was calculated using the ΔΔCT method. **(D)** To evaluate the presence of NETs in the tumor microenvironment of mice treated with the IL-1R antagonist, the levels of citrullinated histone (H3cit) in 4T1 (*n* = 10) and anakinra-treated 4T1 (*n* = 7) tumors were investigated by western blotting. The right panel shows the quantification of the H3cit protein level normalized to that of β-actin. Each dot represents one individual mouse. Values represent the mean ± SD. ^*^P < 0.05 and ^**^P = 0.01; n.s., non-significant, unpaired two-tailed Student's *t*-test.

Considering that the IL-1β/G-CSF inflammatory axis might be involved in the increased propensity for NET formation, we treated the tumor-bearing mice with the commercially available, selective inhibitor of IL-1R, anakinra (40). As shown in Figure 3A, 4T1 tumor-bearing mice treated with anakinra (10 mg/kg/day from day 14 until day 21) exhibited significant reductions in both tumor growth and tumor weight (Figure 3B) compared with tumor-bearing mice that did not receive anakinra treatment. *In vitro* assays showed no direct cytotoxic or cytostatic effects of the IL-1R antagonist on the 4T1 cells (Supplementary Figure 3). Remarkably, anakinra did not affect the growth of 67NR tumors (Figures 3C,D), possibly reflecting the minor participation of the IL-1β/G-CSF axis in 67NR tumor progression. We further analyzed the impact of blocking IL-1R in the 4T1 tumor microenvironment. Although the infiltration of neutrophils (Ly6G^+^ CD11b^+^ cells) into the tumor mass was not affected by treatment with anakinra (Figure 4A and Supplementary Figure 4), a significant reduction in the accumulation of VEGF^+^ neutrophils was observed (Figure 4B). In addition, the blockade of IL-1R significantly reduced the G-CSF mRNA levels in 4T1 tumors (Figure 4C). In contrast, the diminished expression of G-CSF was not sufficient to significantly alter the citrullinated histone levels in the tumor mass, suggesting no effect toward NETs formation in the tumor microenvironment (Figure 4D).

### Inhibition of IL-1R Decreases Circulating G-CSF and Inhibits Venous Thrombosis in 4T1 Tumor-Bearing Mice

**Figure 5:**
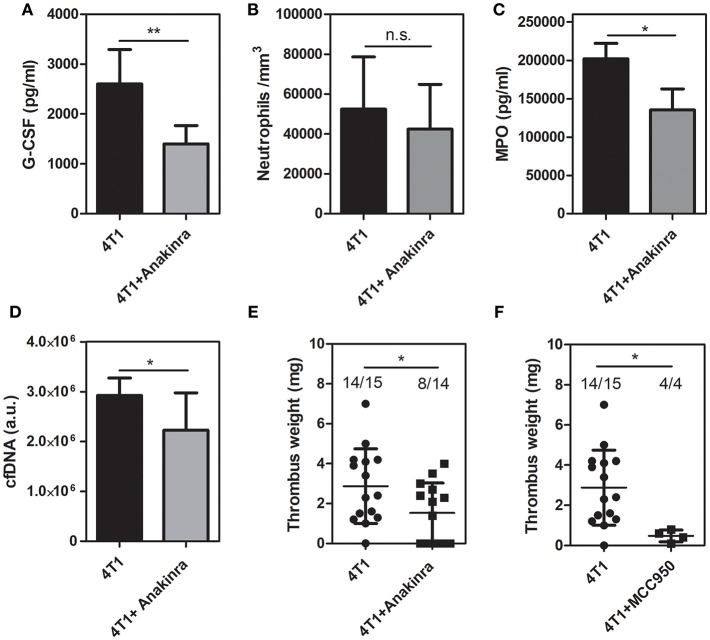
Inhibition of IL-1R decreases DVT in 4T1 tumor-bearing mice. **(A)** Systemic levels of G-CSF in 4T1 tumor-bearing mice (black bars, *n* = 5) and 4T1 tumor-bearing mice treated with anakinra (gray bars, *n* = 6) were assessed by a specific ELISA kit as described in section Materials and Methods. **(B)** The neutrophil count in mice with 4T1 (black bars, *n* = 11) or anakinra-treated 4T1 (gray bars, *n* = 7) tumors was evaluated as described in section Materials and Methods. Two systemic markers of NETs, MPO **(C)** and cfDNA **(D)**, were analyzed in 4T1 tumor-bearing mice (black bars, *n* = 6) and 4T1 tumor-bearing mice treated with the IL-1R antagonist (gray bars, *n* = 6) as described in section Materials and Methods. For systemic analysis, the blood was collected 21 days after the tumor cell inoculation. **(E)** Venous thrombosis was evaluated with stasis of the inferior vena cava for 6 h in 4T1 tumor-bearing mice (•, *n* = 15) and 4T1 tumor-bearing mice treated with anakinra (■, *n* = 14). **(F)** Alternatively, 4T1 tumor-bearing mice (•, *n* = 15) were treated with MCC950 (20 mg/kg/day, s.c.) (■, *n* = 4) for 8 days, between day 14 and day 21 after the tumor cell inoculation. Each dot represents one individual mouse. Values represent the mean ± SD. ^*^P < 0.05 and ^**^P = 0.01; n.s., non-significant, unpaired two-tailed Student's *t*-test.

Since the treatment with anakinra reduced the *G-CSF* levels in the tumor mass, we further evaluated the peripheral effects of the IL-1R antagonist. As observed in the tumor microenvironment, treatment of 4T1 tumor-bearing mice with anakinra caused a significant reduction in the plasma levels of G-CSF (Figure 5A) although no significant changes were observed in the circulating neutrophil (Figure 5B), monocyte or lymphocyte counts (Supplementary Table 2). The plasma levels of myeloperoxidase (Figure 5C) and cfDNA (Figure 5D) were significantly reduced in 4T1 tumor-bearing mice treated with the IL-1R antagonist. In contrast, the systemic levels of the DNA-elastase complex, pointed as a more specific marker of NETs formation, were not affected by treatment with anakinra (Supplementary Figure 5). Remarkably, the blockade of IL-1R abolished the prothrombotic state in 4T1 tumor-bearing mice. As shown in Figure 5E, 4T1 tumor-bearing mice treated with anakinra showed a significant reduction in the thrombus weight compared with the 4T1 tumor-bearing mice that did not receive the treatment (1.53 ± 0.40 vs. 2.87 ± 0.48 mg, respectively). Interestingly, we observed a remarkable difference in the frequency of thrombus occurrence in the treated animals (8 out of 14 animals) compared to untreated animals (14 out of 15 animals). Cytosolic molecular complexes called inflammasomes critically modulate the production of IL-1β (41). Here, we evaluated the effect of the NLRP3 inflammasome inhibitor MCC950 (42) on the prothrombotic state in 4T1 tumor-bearing mice. As shown in Figure 5F, treatment with MCC950 decreased thrombus formation in the DVT model.

## Discussion

In this study, we employed a well-established murine breast cancer model to evaluate whether the IL-1β inhibition could attenuate cancer-associated thrombosis. Our data show that 4T1 tumor-bearing mice exhibit elevated expression of G-CSF and IL-1β in the tumor mass, increased neutrophil counts in the peripheral blood and elevation of indirect markers of NET formation in plasma. In turn, mice bearing non-metastatic 67NR tumors showed low expression levels of G-CSF and IL-1β in the tumor mass as well as no elevation in neutrophil counts or NETosis markers. Interestingly, 4T1 tumor-bearing mice but not 67NR-bearing mice exhibited a NET-dependent prothrombotic state. The blockade of IL-1R with the specific antagonist anakinra decreased the primary growth of 4T1 tumors but did not alter 67NR tumor progression. In addition, treatment with the IL-1R antagonist reduced tumor-derived G-CSF levels without interfering with neutrophil counts. Most remarkably, the blockade of IL-1R abolished the prothrombotic state in 4T1 tumor-bearing mice.

Several lines of evidence implicate NETs as crucial players in venous thrombus formation. Herein, we utilized a model of flow restriction-induced DVT that is highly dependent on the NET formation (13, 14, 43). It is proposed that this model closely resembles the histological features of human DVT, in which stenosis-driven hypoxia leads to a proinflammatory endothelial phenotype and the recruitment of innate immune cells, predominantly neutrophils and monocytes (14, 44). Thus, thrombus formation in this model relies on slow fibrin deposition and the accumulation of leukocytes and platelets (14). Previous studies have shown that neutrophil depletion or treatment with DNase I severely impacts thrombus formation in this model (13, 14). In this context, we observed that treatment with DNase I significantly attenuated thrombus formation in 4T1 tumor-bearing mice. PAD4 is a nuclear enzyme that converts specific arginine residues to citrulline in histone tails, a process that has been shown to be critical during NET formation (37). PAD4-deficient mice exhibit defective thrombus formation in the stenosis-induced DVT model (38). Herein, we demonstrated that GSK484, a reversible inhibitor of PAD4 (39), significantly reduced thrombus formation in 4T1 tumor-bearing mice, although to a lesser extent than DNase I. Overall, it remains to be determined whether preventing the formation of NETs or degrading NETs could efficiently prevent cancer-associated thrombosis in human patients.

The role of infiltrated neutrophils in the tumor microenvironment is still controversial. Sagiv et al. (45) employed several tumor models, including 4T1 breast cancer cells, to propose that neutrophils may acquire two different phenotypes during tumor progression: N1, which is mostly antitumoral, and N2, which is predominantly immunosuppressive and favors tumor growth. In this study, we observed that the treatment of 4T1 tumor-bearing mice with the IL-1R antagonist had little impact on Ly6G^+^ CD11b^+^ cell infiltration of the tumor mass. On the other hand, we found a significant decrease in the Ly6G^+^ CD11b^+^ VEGF^+^ cell population, suggesting that IL-1β supports the polarization of neutrophils to the N2 phenotype. Accordingly, Andzinski et al. (46) have demonstrated that neutrophils polarized into the N2 phenotype display an increased propensity to release NETs in tumor-bearing mice.

G-CSF treatment in humans has been associated with the elevation of markers of systemic NET formation (47) and increased G-CSF levels have been previously correlated with NET-associated microthrombosis in cancer patients (17, 27). More recently, biomarkers of the systemic formation of NETs were associated with the occurrence of venous thromboembolism in certain cancer types (18). The 4T1 murine breast cancer model is characterized by severe neutrophilia as a consequence of the high G-CSF levels secreted by the neoplastic cells (17). Indeed, our data showed that the *G-CSF* expression levels, either in cultured 4T1 cells or in the tumor mass, were significantly higher than those in 67NR cells *in vitro* or *in vivo*, respectively. *In vitro*, G-CSF has previously been shown to stimulate neutrophils to produce NETs (26), a mechanism that has been correlated with NET-mediated metastasis in the 4T1 breast cancer model. In this context, the extremely high plasma concentrations of G-CSF observed in 4T1 tumor-bearing mice but not in 67NR-bearing mice may contribute to the exacerbation of NET release during thrombus formation in the DVT model.

In addition to G-CSF, IL-1β has also been described as a potent inducer of NET formation *in vitro* (48). There is also evidence of an increased IL-1β expression during venous thrombus development, a process that was regulated by the transcriptional activity of hypoxia-inducible factor-1 alpha (HIF-1α). In addition, the genetic blockade of HIF-1α expression or IL-1β production decreased thrombus formation in a flow restriction-induced thrombosis model in rats (49). Researchers have also found that individuals who develop venous thrombosis at high altitudes exhibit increased expression levels of IL-1β in the peripheral blood mononuclear cells. As observed for G-CSF, our data showed that the IL-1β expression levels, either in cultured 4T1 cells or in the tumor mass, were substantially higher compared to those in 67NR cells *in vitro* or *in vivo*, respectively. Most remarkably, we demonstrated that the pharmacological blockade of IL-1R significantly attenuated thrombus formation in 4T1 tumor-bearing mice.

In the context of primary tumor growth and metastasis, IL-1β has been associated with the infiltration of myeloid-derived suppressor cells (MDSCs) and tumor-associated macrophages in the tumor microenvironment (50, 51). Pharmacological or genetic blockade of IL-1R decreases MDSC accumulation and inhibits tumor growth and metastasis in murine breast cancer models. Accordingly, we also observed a significant reduction in 4T1 primary tumor growth in animals treated with the IL-1R antagonist, while no differences were observed in the progression of 67NR tumors. We have also observed that treatment with the IL-1R antagonist decreases *G-CSF* expression in the tumor mass. Welte et al. (51) have shown that silencing G-CSF in 4T1 cells slows tumor progression and decreases MDSC accumulation in the tumor microenvironment.

An inflammasome is a multiprotein complex expressed primarily in myeloid cells, which regulates the generation of IL-1 family proteins, such as IL-1β and IL-18, and forms a caspase-1-activating platform leading to the production of proinflammatory cytokines (41). Targeting the NLRP3 inflammasome reduces tumor progression in different mouse models, including melanoma, breast and oral squamous cell carcinoma models (52). In addition, NLRP3 potentiates venous thrombosis in response to hypoxia upon IL-1β production. Herein, we demonstrated that the selective NLRP3 inhibitor MCC950 (42) decreased thrombus formation in 4T1 tumor-bearing mice.

**Figure 6:**
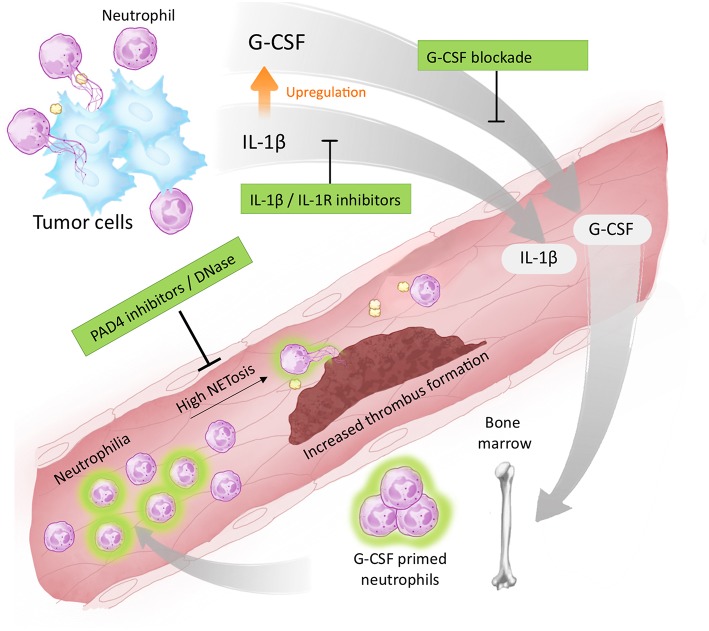
Proposed targets for antithrombotic therapy in NET-dependent thrombosis. The development of a thrombotic state in cancer patients is dependent on tumor-induced systemic alterations. One possible mechanism involves the elevation of circulating levels of IL-1β and G-CSF as well as neutrophilia. G-CSF acts in an endocrine fashion, stimulating the bone marrow to produce and export neutrophils to the bloodstream. G-CSF (and possibly IL-1β) prime neutrophils toward NETosis facilitating the establishment of a NET-dependent prothrombotic state. Blockade of IL-1β expression, or signaling through IL-1R, reduces G-CSF levels attenuating the thrombus formation. Therapies targeting NETs formation (such as PAD4 inhibitors) or destroying already formed NETs (such as DNase I) have also been proposed to attenuate cancer-associated thrombosis.

Taken together, our study demonstrates that IL-1β is a targetable factor that drives cancer-associated thrombosis, possibly by modulating G-CSF production and facilitating NET formation (Figure 6). We have also found evidence that long-term blockade of IL-1R impacts neutrophil polarization, thus contributing to a reduction in the systemic NET release. Therefore, our results demonstrate that blockade of IL-1R decreases G-CSF levels and attenuates the prothrombotic state in tumor-bearing mice. Targeting the IL-1β/G-CSF axis might be relevant in certain cancer types that rely on NET-dependent prothrombotic states.

## Ethics Statement

This investigation was conducted in accordance with ethical standards and according to the Declaration of Helsinki and national and international guidelines and has been approved by the institutional animal care and use committee (IACUC) of the Federal University of Rio de Janeiro. The animal procedures were approved by the IACUC under protocol number CEUA102/16.

## Author Contributions

TG performed and analyzed the experiments, wrote the manuscript, and designed the study. CV, ALL, DM, AR, and BG performed and analyzed the experiments. ACL, D-BH, and EM analyzed the data and participated in the manuscript preparation. RM provided the hypothesis question, wrote the manuscript, designed the study, and analyzed the data. All authors read and approved the final version of the manuscript.

### Conflict of Interest Statement

The authors declare that the research was conducted in the absence of any commercial or financial relationships that could be construed as a potential conflict of interest.
