# Supplementary material for: IL-1β Blockade Attenuates Thrombosis in a Neutrophil Extracellular Trap-Dependent Breast Cancer Model
Source: Front Immunol. 2019 Sep 4;10:2088. doi: 10.3389/fimmu.2019.02088 (PMC6737452; doi:10.3389/fimmu.2019.02088)
Supplement: Supplementary file 1 [file Table_1.docx]

Supplementary Material

IL-1β drives cancer-associated thrombosis by supporting neutrophil extracellular trap formation

**Tainá Gomes^1^, André L. Lourenço^1^, Carolina B. S. Várady^1^, Araci M. R. Rondon^1^, Ana C. Leal^1^, Emiliano H. Medei^2^, Robson Q. Monteiro^1*^.**

*** Correspondence:** Robson Q Monteiro: [robsonqm@bioqmed.ufrj.br](mailto:robsonqm@bioqmed.ufrj.br)

# Supplementary tables

**Supplementary Table 1: Hematological analysis in 4T1 and 67NR tumor-bearing mice**

| **Hematological parameter** | **Control (n=12)** | **67NR (n=5)** | **4T1 (n=11)** |
| --- | --- | --- | --- |
| **Blood cells (millions/mm^3^)** | 7.85 ± 0.65 | 6.2 ± 1.51 ^*^ | 7.75 ± 1.17 ^n.s.^ |
| **Hemoglobin (g/dl)** | 12.99 ± 1.10 | 10.42 ± 2.31 ^n.s.^ | 12.84 ± 2.03 ^n.s.^ |
| **Hematocrit (%)** | 38.16 ± 3.14 | 29.88 ± 7.35 ^n.s.^ | 37.54 ± 5.68 ^n.s.^ |
| **Leukocytes (/mm^3^)** | 5145 ± 2680 | 2512 ± 873.2 ^n.s.^ | 70590 ± 36903 ^*** ###^ |
| **Neutrophils (/mm^3^)** | 2414 ± 1843 | 1820 ± 1041 ^n.s.^ | 52391 ± 26275 ^***^ ^##^ |
| **Lymphocytes (/mm^3^)** | 2371 ± 1484 | 513 ± 228 ^n.s.^ | 11652 ± 10339 ^** #^ |
| **Monocytes (/mm^3^)** | 267 ±226 | 160 ± 115 ^n.s.^ | 5584 ± 4110 ^*** ##^ |
| **Platelets (/mm^3^)** | 336500 ± 124531 | 361800 ± 46149 ^n.s.^ | 460727 ± 122163 ^*^ |

4T1 and 67NR tumor cells (5 × 10^5^) were orthotopically injected into the mammary fat pads of female BALB/c mice. Blood was collected for hemogram analysis 21 days after the tumor cell inoculation as described in the Materials and Methods section. The following parameters were analyzed: blood cells, hemoglobin, hematocrit, leukocytes, neutrophils, lymphocytes, monocytes and platelets. Values represent the mean ± SD. Compared to control, * P< 0.05; ** P< 0.001; *** P< 0.0001; Compared to 67NR, # P< 0.05; ## P< 0.001; ### P< 0.0001; n.s., nonsignificant; analysis of variance (ANOVA) followed by **Tukey's** post hoc test.

**Supplementary Table 2: Hematological analysis of 4T1 tumor-bearing mice treated with anakinra.**

| **Hematological parameter** | **4T1 (n=11)** | **4T1 + Anakinra (n=7)** |
| --- | --- | --- |
| **Blood cells (millions/mm^3^)** | 7.75 ± 1.17 | 7.08 ± 1.319 ^n.s.^ |
| **Hemoglobin (g/dl)** | 12.84 ± 2.03 | 10.93 ± 2.16 ^n.s.^ |
| **Hematocrit (%)** | 37.54 ± 5.68 | 32.83 ± 7.36 ^n.s.^ |
| **Leukocytes (/mm^3^)** | 70590 ± 36903 | 54086 ± 23697 ^n.s.^ |
| **Neutrophils (/mm^3^)** | 52391 ± 26275 | 42419 ± 22385 ^n.s.^ |
| **Lymphocytes (/mm^3^)** | 11652 ± 10339 | 8004 ± 2820 ^n.s.^ |
| **Monocytes (/mm^3^)** | 5584 ± 4110 | 3104 ± 1754 ^n.s.^ |
| **Platelets (/mm^3^)** | 460727 ± 122163 | 367143 ± 19013 ^n.s.^ |

4T1 tumor cells (5 × 10^5^) were orthotopically injected into the mammary fat pads of female BALB/c mice. Treatment with 10 mg/kg/day (s.c.) anakinra was performed for 8 days, between D 14 and D 21. Blood was collected for hemogram analysis 21 days after the tumor cell inoculation as described in the Materials and Methods section. The following parameters were analyzed: blood cells, hemoglobin, hematocrit, leukocytes, neutrophils, lymphocytes, monocytes and platelets. Values represent the mean ± SD. *P < 0.05, **P = 0.01 and ***P = 0.001; n.s.= nonsignificant, unpaired two-tailed Student’s t-test.

# Supplementary figures legends

**Supplementary Figure 1: Expression of H3cit in 4T1 tumor cells.** Complete scanned gels for western blots shown in Figure 1E. **(A)** Gel for H3cit expression and **(B)** β-actin expression.

**Supplementary Figure 2: Treatment with DNase I reduces thrombus formation in the 4T1 model, even after 6 hours of flow restriction.** Venous thrombosis was evaluated with stasis of the inferior vena cava for 6 hours. Figure show the results obtained for 4T1 tumor-bearing mice (circles, n=15) or 4T1 tumor-bearing mice were treated with DNase 1 (50 micrograms, i.v.) (squares, n=9) just before the procedure to induce stasis of the inferior vena cava for 6 hours. Each dot represents one individual mouse. Values represent the mean ± SD. **P = 0.01, unpaired two-tailed Student’s t-test.

**Supplementary Figure 3: Anakinra exhibits no cytotoxic or cytostatic effects on 4T1 cells. (A)** The cell viability of 4T1 cells was analyzed by an MTT assay. The cells were treated with 0.1, 10 or 100 μg anakinra for 24, 48 or 72 hours. **(B)** The proliferation of cells treated with anakinra (100 micrograms) or left untreated was measured by CFSE fluorescence after a period of 24-72 hours. n.s= nonsignificant, unpaired two-tailed Student’s t-test.

**Supplementary Figure 4: Treatment with the IL-1R antagonist has little impact on total tumor-associated neutrophils.** A dot plot of the infiltration of neutrophils in 4T1 (n=6) and anakinra-treated 4T1 (n=6) tumors is shown. The tumors were digested into single cells and assessed for Ly6G^+^ CD11b^+^ cells via flow cytometry.

**Supplementary Figure 5: Systemic levels of DNA-elastase complex.** Figure show the DNA-elastase complex levels measured in the plasma of control (circles, n = 8), 4T1 (triangles, n = 8) and anakinra-treated 4T1 (inverted triangles, n = 4) tumor-bearing mice. Plasma was obtained 21 days after tumor cell inoculation. DNA-elastase complex levels were assessed by a specific ELISA kit as described in Materials and Methods section. *P < 0.05; n.s.= nonsignificant, unpaired two-tailed Student’s t-test.

**
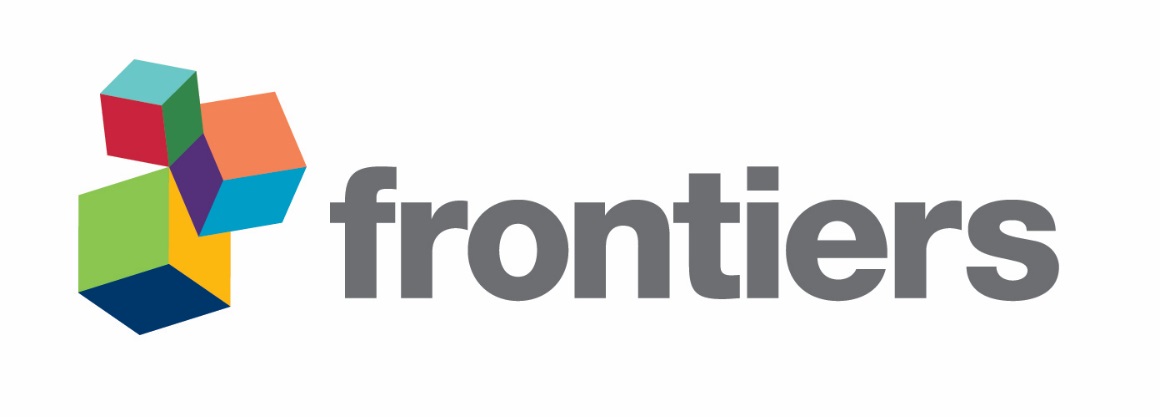
**
