# Supplementary figures and images for: IL-1β Blockade Attenuates Thrombosis in a Neutrophil Extracellular Trap-Dependent Breast Cancer Model
Source: Front Immunol. 2019 Sep 4;10:2088. doi: 10.3389/fimmu.2019.02088 (PMC6737452; doi:10.3389/fimmu.2019.02088)

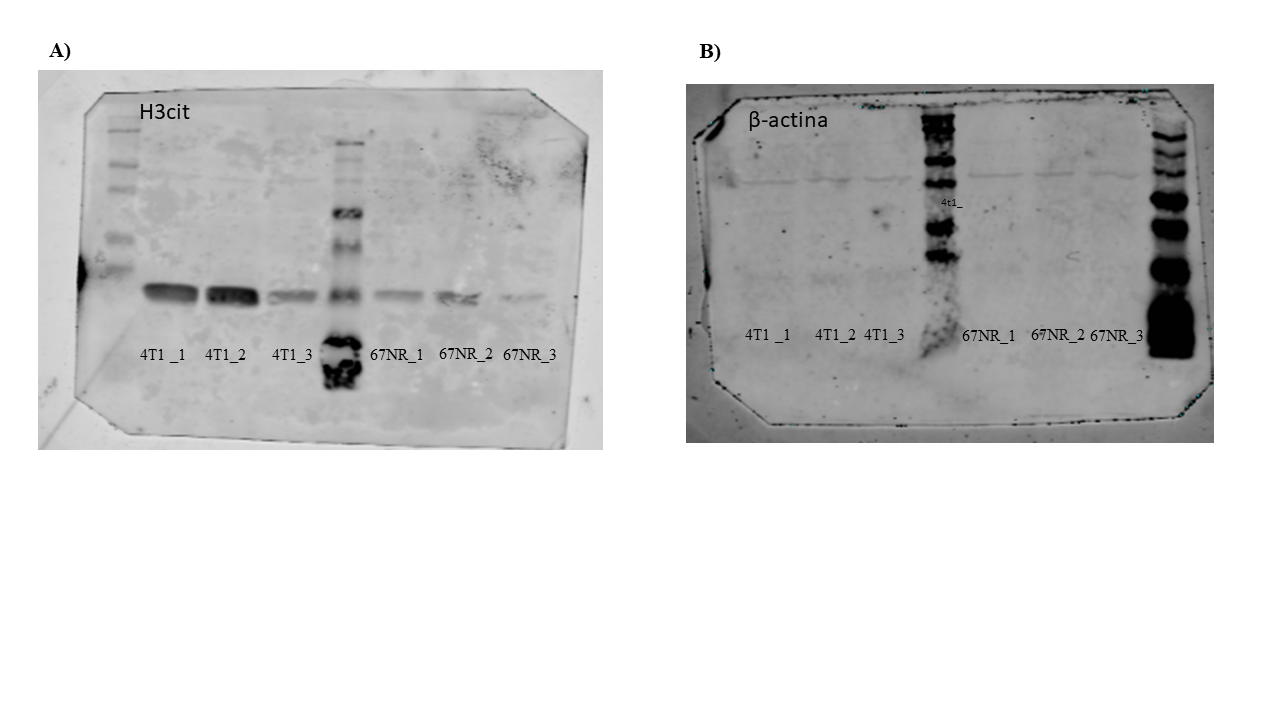

Supplement: Supplementary file 2 [file Image_1.tif]

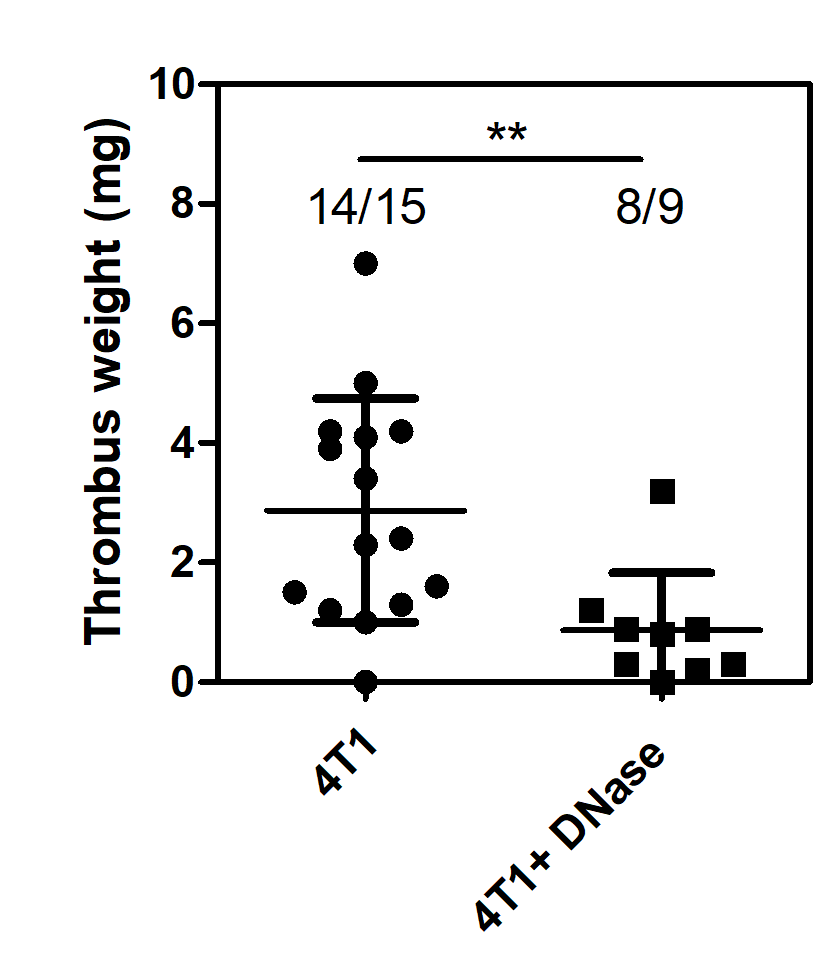

Supplement: Supplementary file 3 [file Image_2.tif]

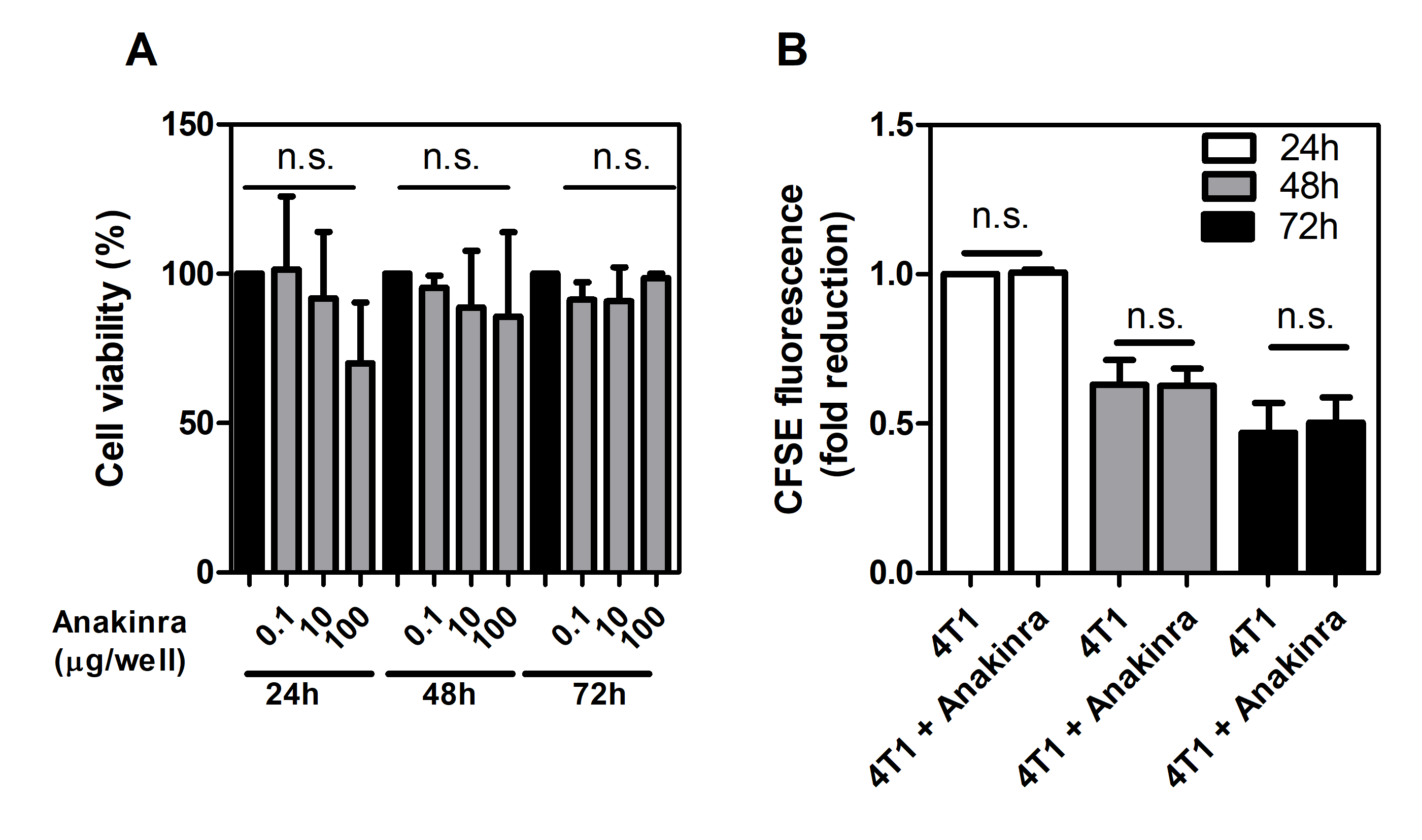

Supplement: Supplementary file 4 [file Image_3.tif]

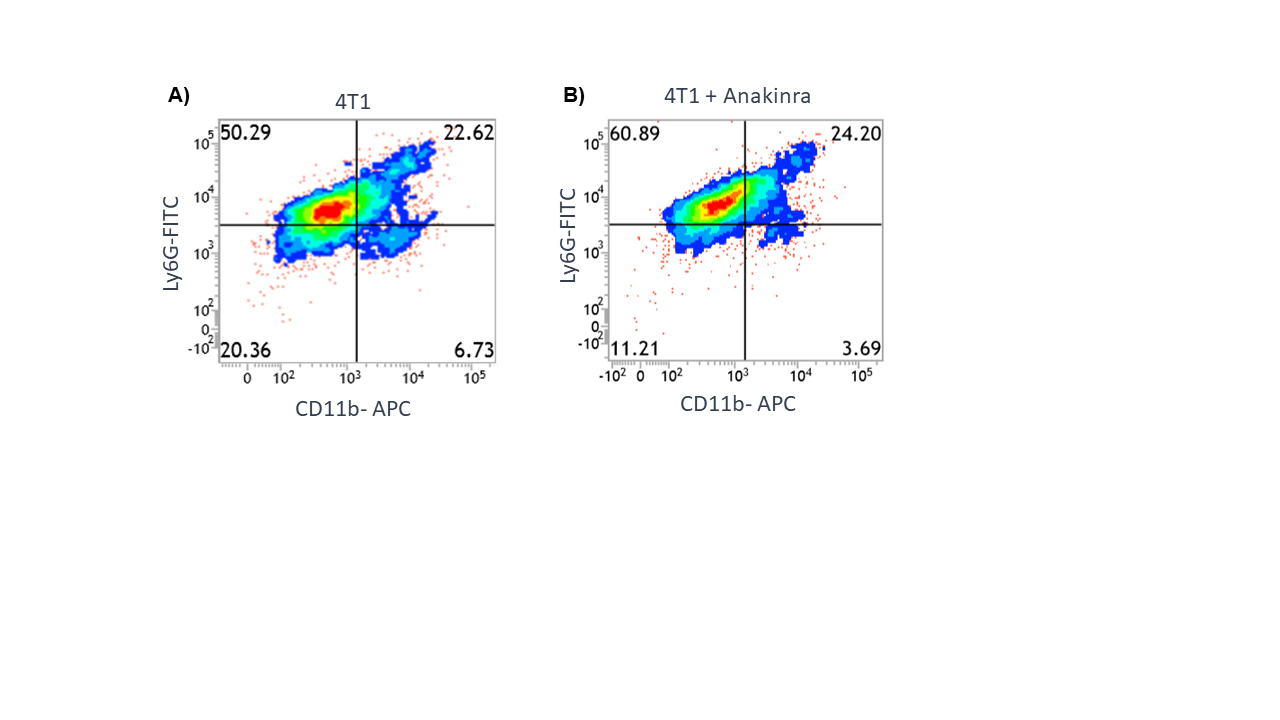

Supplement: Supplementary file 5 [file Image_4.tif]

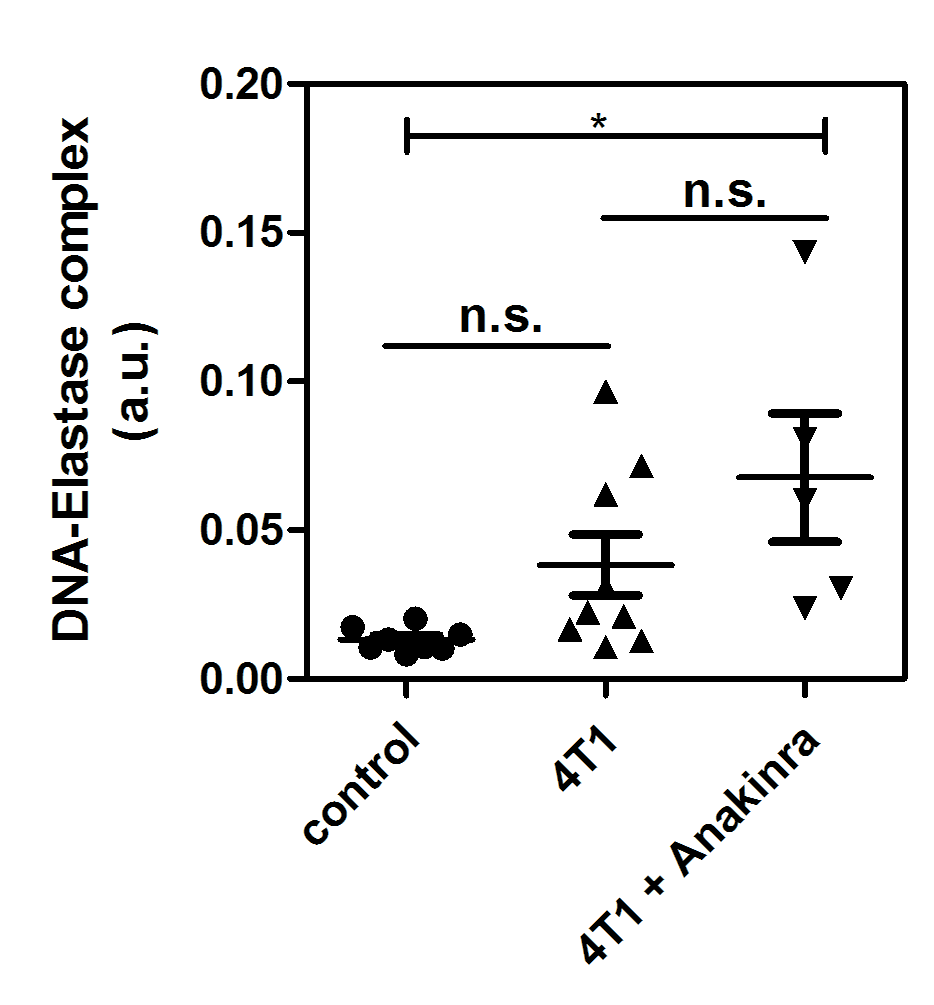

Supplement: Supplementary file 6 [file Image_5.tif]
